# Construction and Validation of a Cell Cycle-Related Robust Prognostic Signature in Colon Cancer

**DOI:** 10.3389/fcell.2020.611222

**Published:** 2020-11-16

**Authors:** Zhiyuan Zhang, Jingwen Chen, Shichao Zhu, Dexiang Zhu, Jianmin Xu, Guodong He

**Affiliations:** ^1^Department of General Surgery, Zhongshan Hospital, Fudan University, Shanghai, China; ^2^Department of Cardiac Surgery, Shanghai Institute of Cardiovascular Diseases, Zhongshan Hospital, Fudan University, Shanghai, China

**Keywords:** cell cycle, colon cancer, signature, molecular subgroups, overall survival

## Abstract

Colon cancer is one of the most common cancers, great progress was taken place in the treatment of colon cancer, however, the prognostic assessment system remains lagging. Cell cycle plays a vital role in the whole procedure of cancers. In this study, we firstly identified cell cycle-related genes specific in colon cancer. Functional enrichment analysis proved our analysis reliable. Furthermore, we constructed a robust signature based on the cell cycle-related genes. The AUC of the signature to predict the overall survival was 0.808, 0.807, and 0.831 of AUC at 1, 3, and 5 years, respectively. Internal and external validation proved the signature efficient. The 9 genes involved in the signature also showed a great job in molecular subgrouping which indicated the significant value of the 9 genes for further experimental research. In conclusion, the present research provided a novel robust signature predicting the prognosis of colon cancer.

## Introduction

Colon cancer is a common cancer type that owns high morbidity and mortality in the world. Nowadays, colorectal cancer ranks the second place of the mortality among all the cancers. Moreover, the age for people diagnosed with colon cancer tends to be younger than the previous, which indicates that the incidence is rising in young people (Siegel et al., 2020).

Owing to the great advance in the treatment of colon cancer including diverse novel surgical technology and different kinds of treatment specific for different kinds of patients, mortality reliefs much than before. However, the progress of the method to assess the prognosis of patients is far inferior to the progress of the treatment of colon cancer. A robust method that can better estimate the patients’ prognosis is also essential and can help to take an early intervention to the patients which may have a bad prognosis. At present, the most common method to estimate the prognosis of colon cancer patients is Tumor-Node-Metastasis (TNM) staging and pathological grading system, yet the effects are just passable (Dienstmann et al., 2017). On the other hand, it is urgent and crucial to establish a novel way to better predict the prognosis of colon cancer patients.

The recently great progressed high-throughput techniques including RNA-seq and microarray provides researchers deep insight into the role of molecular biomarkers in cancers (Zhang et al., 2018a,b; Qian et al., 2019). Furthermore, when combined with matched clinical information, this kind of technology also gives us a lot of inspiration in predicting the prognosis of patients. Many great signatures based on large sample size contributed a lot to the assessment of the prognosis of colon cancer patients (Zhang et al., 2020a,b,c). However, few of them are widely used in clinical practice mainly due to this signature still have defects which may because the original gene set cannot include the entire process of cancer.

Cell cycle is involved in almost every phase of the progression of cancer because the proliferation of cancer cells is mainly regulated by the cell cycle. However, the relevant signature based on the cell cycle is rare and remains a lack in the aspect of colon cancer. In this research, we identified specific cell cycle-related genes in colon cancer and constructed an efficient signature and further built a nomogram which combined the signature and some significant clinical features in order to predict the prognosis of patients who were diagnosed with colon cancer.

## Materials and Methods

### Acquisition and Processing of Raw Data

Original data including microarray data and matched clinical data was downloaded from the website of database Gene Expression Omnibus (GEO,^1^). Method named Robust Multichip Average was carried out to normalize the raw data (Irizarry et al., 2003). According to the EntrezGeneID, probes were mapped. When more than one probes mapped to only one EntrezGeneID, the mean value was taken for further analysis. In this research, four colon cancer GEO datasets with relevant clinical information were included: GSE39582 (containing 585 patients), GSE17538 (containing 238 patients), GSE29621 (containing 65 patients), GSE39084 (containing 70 patients). GSE39582 which has the largest sample size among the four was defined as the discovery group. The other three datasets were combined as one which was defined as the validation group. The batch effects were balanced through Combat via the package “SVA” in R (Version 3.61).

### Mining of Specific Cell Cycle-Related Genes in Colon Cancer

In order to screen out the cell cycle-related genes specific in colon cancer. The enrichment of cell cycle for each sample in the discovery set was computed through single-sample gene set enrichment analysis (ssGSEA) (Barbie et al., 2009). The reference genes set were retrieved from MSigDB^2^. “KEGG_CELL_CYCLE” filtered from “KEGG gene sets as Gene Symbols” in “c2: curated gene sets” was selected, and “GO_CELL_CYCLE” was also picked from “all GO gene sets as Gene Symbols” in “c5: Ontology gene sets”(Supplementary Table 1). These 2 gene sets were identified as references for ssGSEA algorithm. The Spearman’s correlation of the 2-enrichment scores with each gene was calculated. Then the genes which owned both the absolute correlation >0.3 and *p*-value < 0.01 were defined as colon cancer-specific cell cycle-related genes. ssGSEA was carried out by package “GSVA” in R (Version 3.6.2).

### Survival and Functional Enrichment Analysis

Overall survival-related genes were identified through univariate Cox regression analysis via R (Version 3.62). Functional enrichment analysis was classified into two parts including Gene Ontology (GO) and Kyoto Encyclopedia of Genes and Genomes (KEGG). GO can be detailed divided into the biological process (BP), cellular component (CC), and molecular function (MF). The GO and KEGG analysis were conducted by package “clusterProfiler” in R (Version 3.62) and the criterion for significant results was *q*-value < 0.05 and *p*-value < 0.05 (Yu et al., 2012).

### Construction and Validation of the Signature

The GSE39582 set was randomly divided into two groups at the ratio of 1:1 which named internal training group and internal validation group. Least absolute shrinkage and selection operator (LASSO) was conducted in the internal training group to identify the variates. The variates obtained from LASSO were included to construct the signature via multivariate cox regression. Time-dependent receiver operating characteristic curve (tROC), risk-score analysis and Kaplan-Meier (KM) analysis was utilized to assess the accuracy of the model in the internal training set and were further validated in the internal validation set and external validation set. In order to prove the signature as an independent risk factor, the signature combined with other widely accepted clinical risk factors was further calculated by univariate and multivariate Cox regression analysis. The clinical features which were indicated to have significant contributions to survival were combined with the signature to build a nomogram, calibration analysis was then carried out. The process above were all conducted by R (Version 3.6.2), *p*-value < 0.05 was defined as statistically significant.

### Identification of the Subtypes and Survival Analysis

The expression matrix of genes that were included in the signature was utilized to identify the molecular subtypes in colon cancer via R package “ConsensusClusterPlus” in R (Version 3.6.2). Survival analysis was carried out and visualized in the individual subtype.

## Results

### Identification of Colon Cancer-Specific Cell-Cycle Related Genes

**FIGURE 1:**
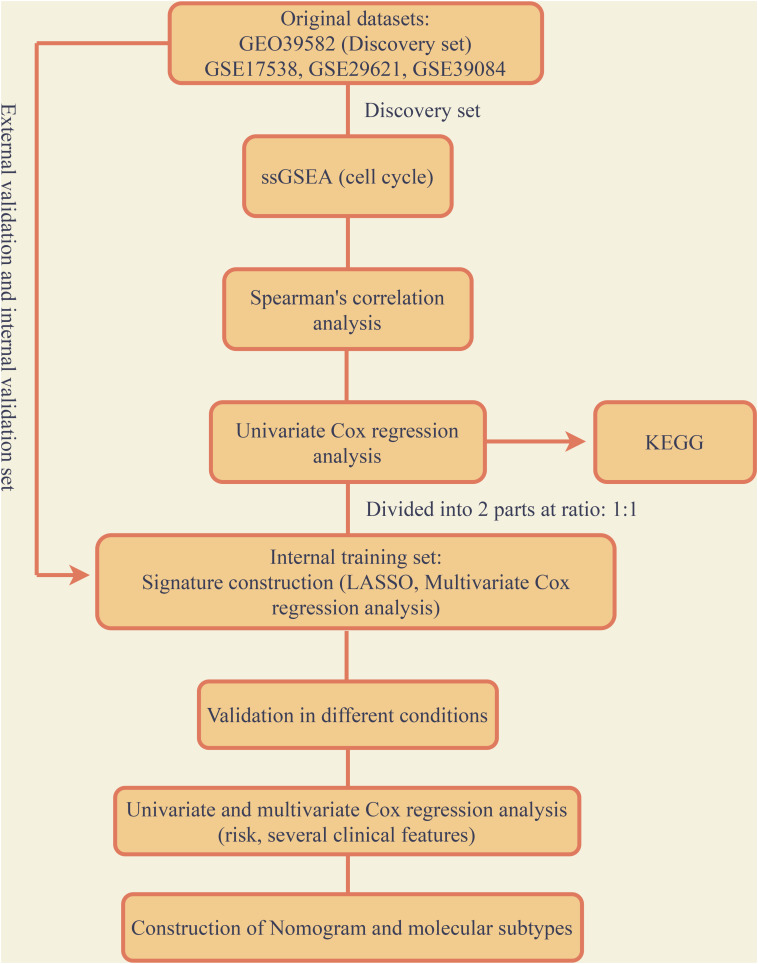
Flow chart of the entire research.

The entire analysis flow was presented in Figure 1. According to the raw microarray data and matched clinical information from GSE39582, we performed ssGSEA and took cell-cycle related gene sets as references. Spearman’s correlation of each gene with cell-cycle enrichment scores was calculated as described in the session of Methods and materials and 2767 genes were identified as colon cancer-specific cell-cycle related genes (Supplementary Table 2). We then conducted univariate Cox analysis to screen out the genes which were significantly correlated with survival based on the cell-cycle related genes (*p*-value < 0.05). Finally, 668 genes were obtained for further analysis (Supplementary Table 3).

### Functional Enrichment Analysis

We performed functional enrichment analysis including GO and KEGG analysis based on the 668 genes mentioned before, the results were presented in Supplementary Figure 1. For KEGG, the most enriched pathways were Cell cycle, DNA replication and Mismatch repair. For the results of GO, it mainly contained three parts including BP, CC, and MF. In terms of BP and DNA replication, chromosome segregation and DNA-dependent DNA replication took the top three places. In terms of CC, chromosomal region, condensed chromosome and condensed chromosome, centromeric region took the top three places. In terms of MF, catalytic activity, action on DNA, catalytic activity, action on RNA and helicase activity took the highest three places. Additionally, cell cycle related functional results were both enriched in GO and KEGG, such as cell cycle DNA replication and cell cycle G1/S phase transition in BP, Cell cycle and p53 signaling pathway in KEGG, these findings demonstrated that the 668 genes identified before enriched in cell cycle and further proved our previous analysis reliable.

### Construction of Cell-Cycle Related Prognostic Signature in Colon Cancer

**FIGURE 2:**
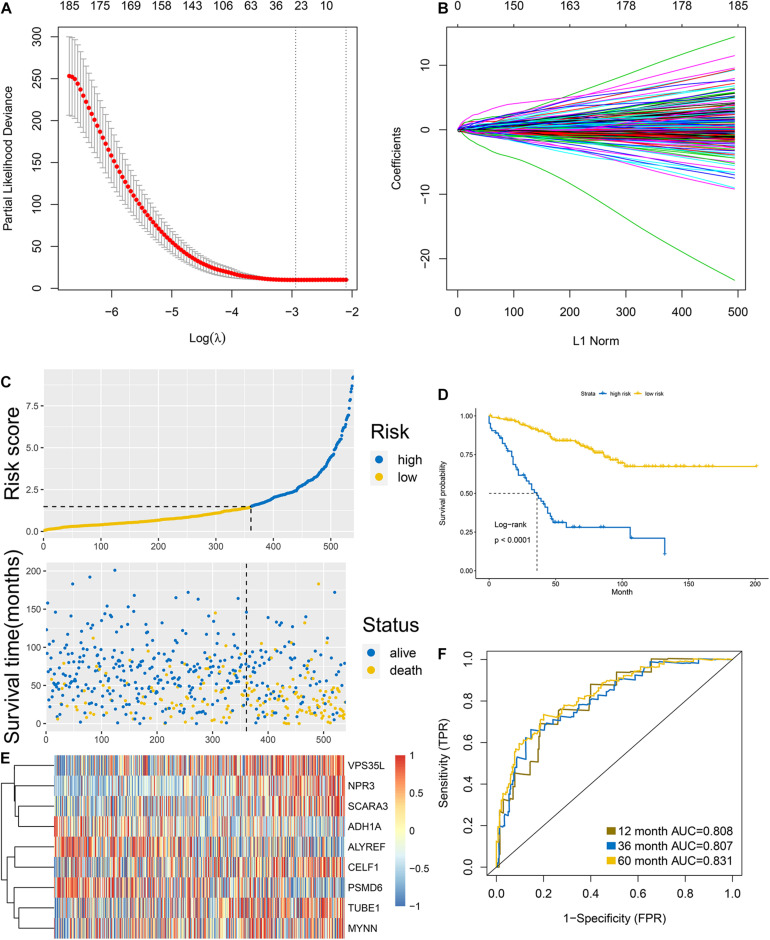
Construction of the signature in the internal training group. **(A,B)** LASSO analysis: the association between deviance and log(λ) **(A)**; the association between coefficients of genes and log(λ). **(C)** Risk score and **(D)** survival analysis of the high and low groups classified by the signature. **(E)** Heatmap of the expression of each gene involved in the signature. **(F)** AUC of the ROC.

The 668 obtained in the previous analysis were selected to construct the cell cycle-related prognostic model to estimate the overall survival of patients. We firstly randomly divided the discovery group into two parts with the ratio of 1:1 that were recognized as internal training and internal validation group, respectively. LASSO regression was then performed based the colon cancer-specific cell cycle-related genes. Cross-validation was carried out (Figure 2A), the log(λ) with the lowest deviance was picked to select the mRNAs whose coefficients were not 0, and 24 genes were included in the signature (Figure 2B). They were PSMD6, TUBE1, MYNN, ALYREF, LYPD6, DHX33, SLC35G1, PA2G4, CCDC134, NUP93, RBBP5, METTL2B, SNRNP25, CELF1, TSPYL2, SCARA3, NPR3, VPS35L, ADH1A, LOXL4, CDK20, HSPA1L, KIF7, and PRRX2. Multivariate Cox regression was conducted to construct the prognostic signature based on the genes retrieved from the LASSO analysis. Finally, 9 genes were included in the prognostic signature, the signature was built as following: risk score = (exp of PSMD6 ^∗^ −2.715) + (exp of TUBE1 ^∗^ 1.538) + (exp of MYNN ^∗^ 1.344) + (exp of ALYREF ^∗^ −0.531) + (exp of CELF1 ^∗^ −2.028) + (exp of SCARA3 ^∗^ 1.278) + (exp of NPR3 ^∗^ 0.734) + (exp of VPS35L ^∗^ 1.1.02) + (exp of ADH1A ^∗^ −0.682). The risk score for individual patients was computed and 1.479 was selected as the cutoff for the high and low-risk group. The results of risk score demonstrated that the model could significantly distinguished the high and low-groups and utilizing the cutoff, the 2 groups differed obviously in survival according to the survival analysis (Figures 2C,D). The expression of each gene in the signature for every patient was shown as heatmap in Figure 2E. The accuracy was assessed by AUC of ROC in Figure 2F, as shown, the AUC was 0.808 at 1 year, 0.807 at 3 year and 0.831 at 5 year, respectively.

### Validation of the Signature in the Internal Validation Group and External Validation Group

**FIGURE 3:**
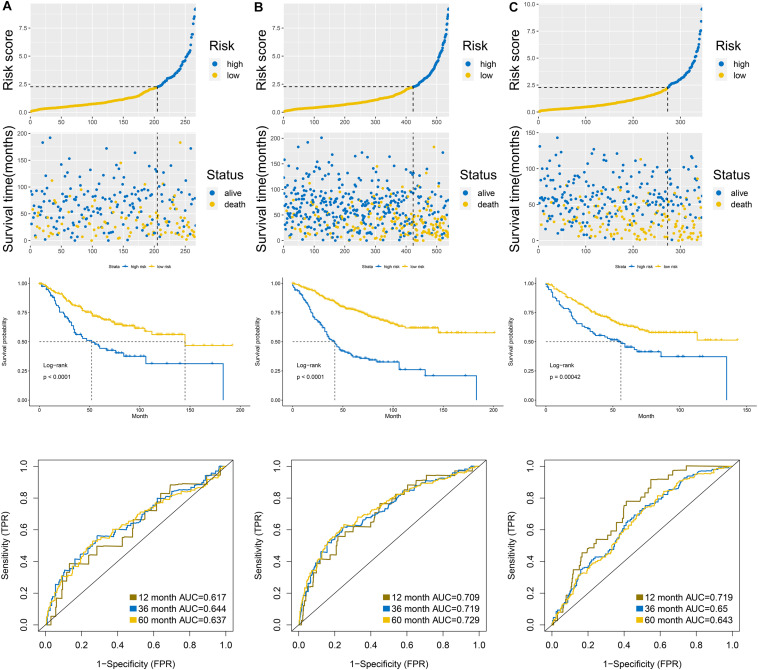
Validation of the signature in the internal validation set **(A)**, entire discovery set **(B)**, and external validation set **(C)**. Upper panel was the risk score analysis, middle panel was the survival analysis, and the down panel was the AUC of the ROC.

**FIGURE 4:**
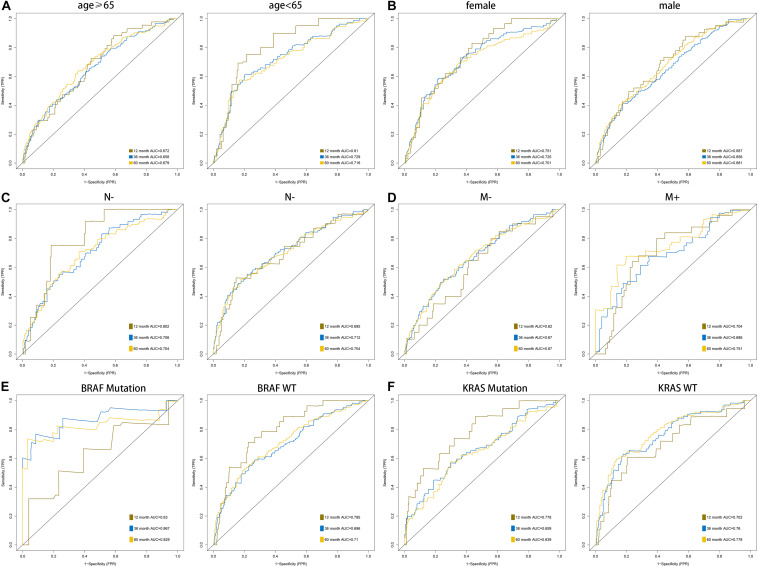
AUC of the ROC that was computed by the signature under diverse situations classified by several clinical features including age **(A)**, gender **(B)**, status of node metastasis **(C)**, status of distant metastasis **(D)**, status of BRAF and status of KRAS **(E,F)**.

**FIGURE 5:**
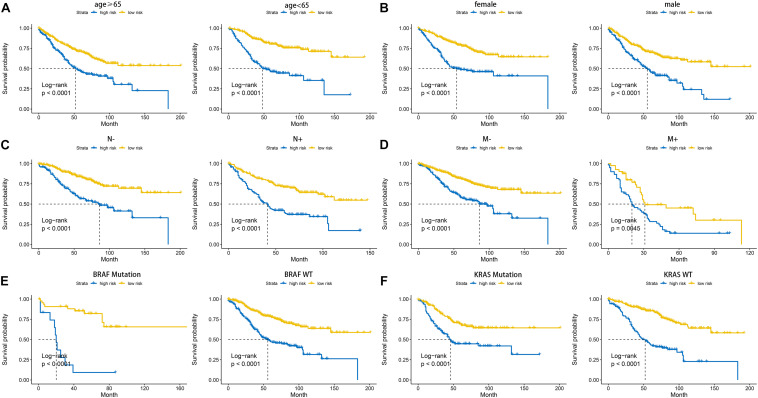
Kaplan-Meier plot of the high and low-risk groups identified by cell cycle-related signature under diverse situations by classifying the patients into different subgroups according to age **(A)**, gender **(B)**, status of node metastasis **(C)**, status of distant metastasis **(D)**, status of BRAF and status of KRAS **(E,F)**.

According to the formula of the signature, we assessed the accuracy of the signature in internal validation set and the external validation set. The risk score analysis, survival analysis and ROC analysis were repeated in the internal validation group, the entire discovery set and the external validation group (Figures 3A–C, internal validation set: left panel, entire discovery set: middle panel and the external validation set: right panel). The model still could distinguish the high and low-risk group with great efficiency and the survival analysis proved the cutoff still worked. The AUC of ROC in different sets further demonstrated that the signature robust. The AUC in the internal validation group was 0.617, 0.644, and 0.636 at 1, 3, and 5 years. The AUC of the entire discovery was 0.708, 0.719, and 0.729 at 1, 3, and 5 years. The AUC in the external validation set was 0.719, 0.65, and 0.643 at 1, 3, and 5 years, respectively. We also analyzed the efficiency of the signature in the entire set under different situations. We classified patients into diverse groups according to different clinical features including age, gender, status of node metastasis, status of distant metastasis, status of BRAF and status of KRAS. The ROC of each group in diverse situations was presented in Figures 4A–F, and the results of survival analysis were shown in Figures 5A–F. These results all indicated that the signature could have great efficiency and keep stable in diverse situations. Moreover, we included some clinical characteristics with the signature into univariate Cox regression analysis. We then screened out the factors which were statically significant in univariate Cox analysis into multivariate Cox analysis, the results all demonstrated that the risk score was an independent risk factor no matter in univariate or multivariate Cox regression analysis (Table 1).

**TABLE 1:** Univariate and multivariate Cox analysis of the signature combined with other clinical features.

Characteristics	HR	HR.95L	HR.95H	*p*-value

Univariate Cox analysis				
Age	1.022209	1.011159	1.033381	7.47E-05
Sex	1.311681	0.989627	1.73854	0.059103
T	1.704863	1.330897	2.183907	2.42E-05
N	1.485482	1.26359	1.746338	1.63E-06
M	4.847453	3.540406	6.637036	7.14E-23
Riskscore	3.008333	2.274627	3.978705	1.15E-14

**Multivariate Cox analysis**				

Age	1.026481	1.015583	1.037496	1.59E-06
T	1.428958	1.103111	1.851056	0.006869
N	1.288663	1.077962	1.540549	0.005367
M	3.495931	2.485858	4.916423	6.28E-13
Riskscore	2.234377	1.671103	2.987511	5.81E-08

### Construction of a Nomogram and Identification of Molecular Subtypes Utilizing the Genes Included in the Signature

**FIGURE 6:**
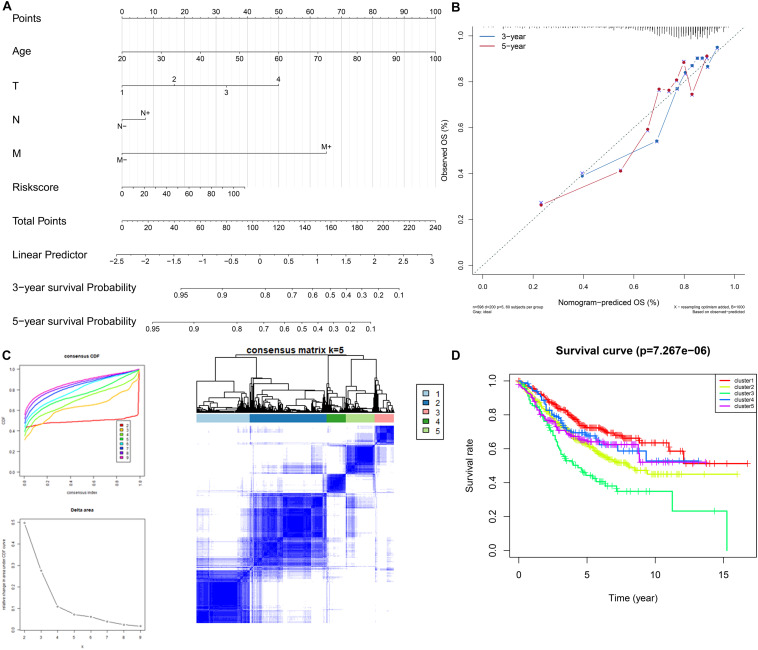
Construction and validation of the nomogram. Details of the nomogram **(A)**, calibration analysis based on the nomogram **(B)**. Molecular subgrouping based on the 9 genes involved in the signature **(C)**: Left panel: Elbow and gap plot for different numbers of subgroups; Right panel: Consensus heatmap of the clusters. Kaplan-Meier plot of the four subgroups **(D)**.

We selected the factors which were both statistically significant after univariate and multivariate Cox analysis to build a nomogram, and calibration analysis was used to assess the efficiency and accuracy. The results were shown in Figures 6A,B. We further used the 9 genes involved in the signature to seek if these genes could classify the patients into different molecular subgroups. The results were considerable, and the molecular subtypes showed different survival outcomes based on survival analysis which also proved the potential clinical contributions of the molecular subtypes classification method (Figures 6C,D). The analysis also implied the 9 genes involved in the signature could have great possibilities to become biomarkers for colon cancer and proved the signature might have a vital role in the clinical contributions.

## Discussion

Colon cancer is one of the most common cancers worldwide. Due to the great advance in the treatment of colon cancer, the mortality decreases in recent years, however, the progress of the system to assess the prognosis of colon cancer lags much. For colon cancer, the main method to assess the outcome of patients is TNM, pathological grading systems, and some other markers such as KRAS status and BRAF status (Yu et al., 2012). But even the patients shared the same TNM may still have diverse outcomes. In the era of precision medicine, the present prognostic system still has a large space to improve in order to estimate the individual outcome for each patient. Moreover, if we can precisely predict the prognosis of individual patients well, the beforehand intervention can be taken to the high-risk patients, and we may even improve the outcomes or the survival of these high-risk patients.

Benefited from the fast progress in high-throughput sequencing in recent years, we can obtain the transcriptome profiling data for every patient. It provided us novel insights into the precise assessment of the prognostic of the individual patient if combined with the relevant clinical data. Based on the technology, many researchers found many vital biomarkers that played significant roles in the process of cancers (Zhang et al., 2018b,c). And many signatures based on the transcriptome profiling data were constructed in recent years (Liu et al., 2019; Tan et al., 2019). However, few of these signatures were utilized in the clinical treatment may due to these signatures were flawed in some terms. The main reason for the deficiency may be the gene sets that were selected to construct the signature could not overall estimate the process of cancers.

Cell cycle is an important biological process that participates in the whole process of cell division. The cell cycle contained the start of cell division to the end of division, it can be detailed interpreted into two parts: inner-phase and division stage. Cell cycle checkpoints which were usually classified as G1 and G2 checkpoints was recognized as an important part in regulating the cell cycle. For most cancer cells, they are defective in G1 checkpoints, because it more common to obtain mutations or other changes of key regulators among G1 checkpoints. Two types of G2 checkpoint responses have previously been identified: DNA damage G2 checkpoint and degrading G2 checkpoint. ATM/CHK2/p53 pathway is the main participant of DNA damage and degradation G2 checkpoint, and the weakening of any G2 checkpoint will lead to chromosome instability. Additionally, cell cycle also regulates the proliferation of cells. Dysregulated cell cycle-related genes can be the root reason for uncontrolled cell proliferation, moreover, uncontrolled cell growth is one of the main causes and the specific features of cancer. On the other hand, cell cycle plays a significant role in the entire process of cancer, many researchers discovered dysregulated genes could affect the development of cancer by influencing the cell cycle (Qian et al., 2018; Tan et al., 2019). However, the researches that built a robust signature based on cell cycle-related genes to predict the prognosis of cancers is rare and remains blank in colon cancer.

In this research, we specifically integrated researched the cell cycle and survival-related genes in colon cancer, 668 genes were identified as cell cycle-related genes. Functional enrichment analysis proved that these 668 genes quite focused on cell cycle. Furthermore, through LASSO and multivariate Cox analysis, we built a signature based on these cell cycle-related genes. The signature showed great efficiency with 0.808, 0.807, and 0.831 of AUC at 1, 3, and 5 years, respectively, in the internal training group. Moreover, the signature was also robust in the internal validation and external validation group. Even under different conditions such as dividing the patients into diverse groups based on different clinical features, the signature still showed great efficiency. In conclusion, the cell cycle-related signature is stable and robust to assess the prognostic outcomes of colon cancer patients. Furthermore, we also included some clinical features which could influence the survival of colon cancers and constructed a nomogram along with the signature for better transform to clinical application.

According to the great efficiency of the signature, we wonder if these genes involved in the signature were important in the process of colon cancer, and we tried to use the expression of the 9 genes to classify colon cancer patients into different molecular subtypes. The result was exciting, we successfully classified patients into 5 subgroups and these five groups showed different survival outcomes which further indicated that the identification of the five subgroups was valuable. The analysis also implied that the 9 genes involved in the signature were of great possibilities for research in colon cancer. PMSD6 was reported to join in numerous cellular processes such as cell cycle, apoptosis, or DNA damage repair (Kanayama et al., 1992). ALYREF is dysregulated in a wide variety of cancer types (Viphakone et al., 2015). CELF1 was identified as a key regulator for melanoma-enriched pro-oncogenic networks (Cifdaloz et al., 2017). SCARA3 was also involved in the signature for estimating the prognosis of glioblastoma (Cao et al., 2019). NPR3 was proved to be inhibited by long non-coding RNA MRCCAT1 and further promoted the metastasis in clear cell renal cell carcinoma (Li et al., 2017). ADH1A was found to associated with proteome and included in HCC metabolic reprogramming. However, our study still had limitations, our signature was constructed based on the training set and further validated in the inner validation and external validation set, the experimental validation is still lack and the signature was not verified in the clinical treatment. In conclusion, our analysis identified specific cell cycle-related genes, and an efficient prognostic signature was constructed in colon cancer. The signature showed great efficacy in predicting the outcomes of colon cancer patients and the 9 genes involved in the signature further presented considerable value in molecular subgrouping. On the other hand, the present research screened out 9 genes that have possibilities as key biomarkers in colon cancer and provided us novel insights into the study of the relation of cell cycle and cancers.

## Data Availability Statement

The datasets presented in this study can be found in online repositories. The names of the repository/repositories and accession number(s) can be found below: https://www.ncbi.nlm.nih.gov/geo/, GSE39582 https://www.ncbi.nlm.nih.gov/geo/, GSE17538 https://www.ncbi.nlm.nih.gov/geo/, GSE29621 https://www.ncbi.nlm.nih.gov/geo/, GSE39084.

## Author Contributions

ZZ, JX, and GH designed and conducted the study. ZZ wrote the article. JC and DZ helped to improve and design the study. SZ helped to improve the writing. All authors contributed to the article and approved the submitted version.

## Conflict of Interest

The authors declare that the research was conducted in the absence of any commercial or financial relationships that could be construed as a potential conflict of interest.

## Footnotes

1 http://www.ncbi.nlm.nih.gov/geo/

2 https://www.gsea-msigdb.org/gsea/index.jsp

## Supplementary Material

Supplementary Figure 1 Functional enrichment analysis of 668 genes that were identified as cell cycle and survival-related genes specific in colon cancer. **(A)** GO analysis including biological process (BP), cellular component (CC), and molecular function (MF). **(B)** KEGG analysis.

Supplementary Table 1 Genesets used as references for cell cycle ssGSEA.

Supplementary Table 2 Details of 2787 genes that were significant correlated with cell cycle in colon cancer.

Supplementary Table 3 Results of univariate Cox analysis of the 668 genes that were defined as cell cycle and survival-related genes in colon cancer.
