# Supplementary figures and images for: Construction and Validation of a Cell Cycle-Related Robust Prognostic Signature in Colon Cancer
Source: Front Cell Dev Biol. 2020 Nov 16;8:611222. doi: 10.3389/fcell.2020.611222 (PMC7701219; doi:10.3389/fcell.2020.611222)

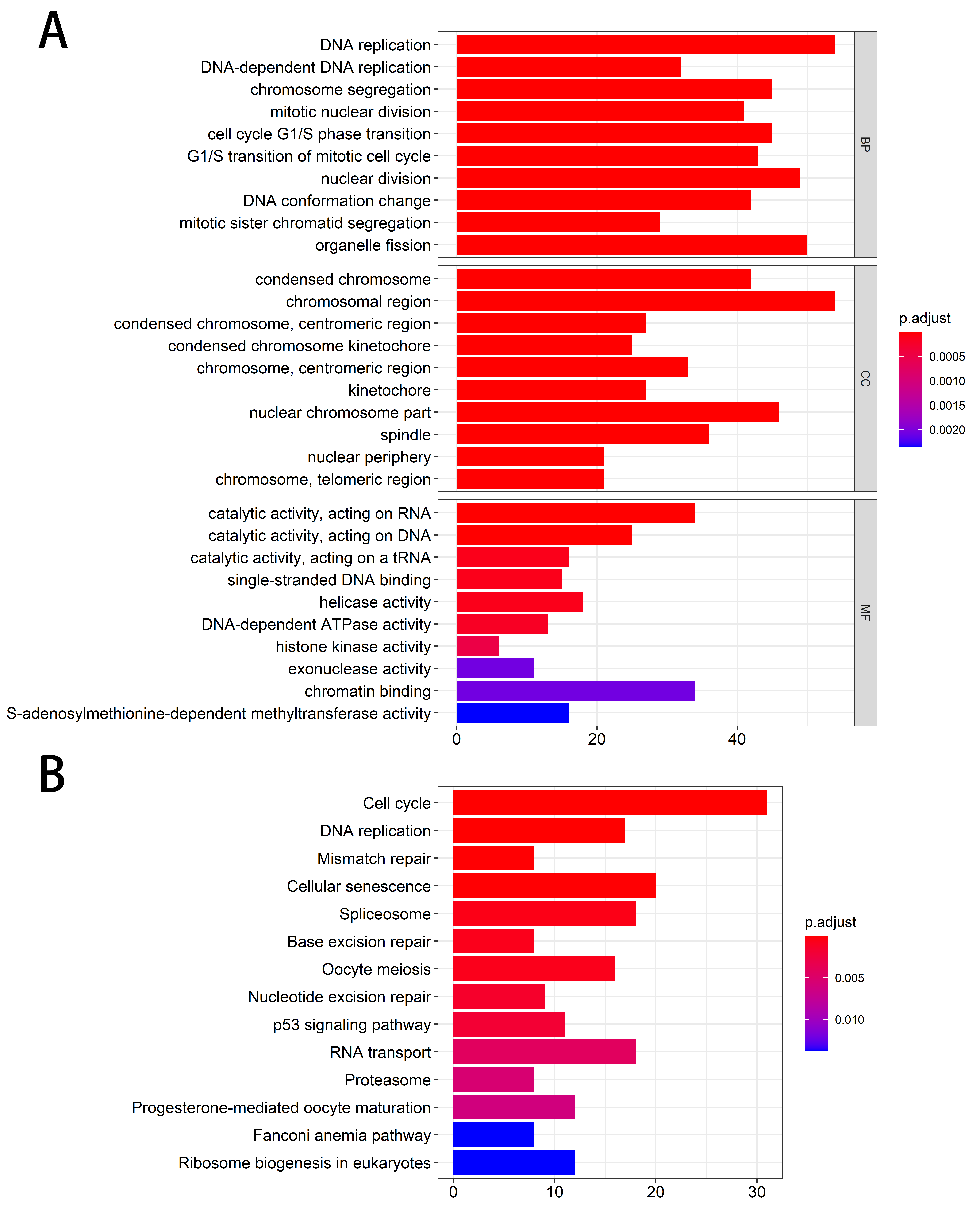

Supplement: Supplementary Figure 1 — Functional enrichment analysis of 668 genes that were identified as cell cycle and survival-related genes specific in colon cancer. (A) GO analysis including biological process (BP), cellular component (CC), and molecular function (MF). (B) KEGG analysis. [file Image_1.TIF]
